# Lactoferrin Levels in the Gastric Tissue of *Helicobacter pylori*-Positive and -Negative Patients and Its Effect on Anemia

**DOI:** 10.1155/2012/214581

**Published:** 2012-03-19

**Authors:** Yaşar Doğan, Tülay Erkan, Zerrin Önal, Merve Usta, Gülen Doğusoy, Fügen Çullu Çokuğraş, Tufan Kutlu

**Affiliations:** ^1^Division of Pediatric Gastroenterology Hepatology and Nutrition, Department of Pediatrics, Cerrahpaşa Medical Faculty, İstanbul University, İstanbul 23119, Turkey; ^2^Fırat Üniversitesi Hastanesi Çocuk Sağ. ve Hast. Anabilim Dalı Elazığ, Turkey; ^3^Department of Medical Pathology, Cerrahpaşa Medical Faculty, İstanbul University, İstanbul 23119, Turkey

## Abstract

*Aim*. To determine gastric tissue lactoferrin (Lf) levels of *Helicobacter pylori-* (Hp-) positive and -negative patients and its effect on anemia. *Methods*. Cases in which initial presentation was of abdominal pain and that were Hp-positive at endoscopy were included. Hp-positive cases and -negative controls were divided into two groups. *Results*. The study included 64 cases (average: 10.2 ± 0.4 years, 39 male and 25 female). Lf levels were subsequently studied on 61 cases. 45 (73.8%) of these were Hp-positive, while 16 (22.2%) were Hp-negative. In Hp-positive cases, mean staining percentages and density of glands in the antral mucosa were 45.5 ± 4.7% and 1.9 ± 0.1, respectively. Hp-negative cases showed significantly different values of 17.8 ± 4.5% and 1.3 ± 0.2, respectively. Hemoglobin and serum ferritin values of Hp-positive cases were 12.7 ± 0.2 g/dL and 32.5 ± 2 ng/mL, but these were comparable with Hp-negative cases (12.6 ± 0.1 g/dL and 30.7 ± 4.4 ng/mL). *Conclusions.* Tissue Lf was significantly higher in Hp-positive cases compared to Hp-negative cases, but no difference was observed between the two groups with regards to hemoglobin and ferritin level. As a result, it is difficult to say that this rise in Lf plays a role in the development of iron deficiency anemia in Hp-positive patients.

## 1. Introduction

*Helicobacter pylori* (Hp) is an important etiologic cause of diseases such as chronic gastritis, duodenal and gastric ulcers, atrophic gastritis, intestinal metaplasia, and gastric lymphoma (Mucosa-associated lymphoid tissue, MALT lymphoma type) [1, 2]. Many studies suggest a relationship between iron deficiency anemia (IDA) and Hp infection, but it is not clear the mechanism by which this occurs [3, 4]. One of the suggested mechanisms occurs through Hp-mediated Lf increase in gastric tissue via neutrophils. Lf captures iron from transferrin. The iron, thus, bound to Lf is in turn picked up by the bacterium, by means of its outer membrane receptors, for its own growth. As *H. pylori* turnover is very rapid, the bacterial iron stores are rapidly lost in the stools, together with the dead bacteria. This mechanism, or at least its template, could explain why an iron supply is no longer available for hemopoiesis, which only enhances *H. pylori* proliferation [5, 6]. The proposed hypothesis is not able to answer why IDA does not develop in all infected subjects. It is possible that the presence of specific bacterium strains and the high needs for iron required by the host under particular conditions could both play a crucial role in the onset of IDA [7]. Therefore, the purpose of this study is to determine the Lf levels in the gastric tissue of Hp-positive and -negative patients and whether this has an effect on anemia.

## 2. Methods

Patients over 4 years of age with recurring abdominal pain of unknown cause who subsequently underwent endoscopy at our hospital's Pediatric Gastroenterology, Hepatology and Nutrition Department have been included in the study. Approval was obtained from the hospital's ethical committee prior to this study. Before the patients were examined, their families were informed within the frame of the Helsinki report, and their signatures were taken on an informed consent form. Following record of age, gender, weight, height, and clinical details, all cases underwent an upper GIS endoscopy aimed at diagnosis. During the endoscopy, biopsy samples were taken from the gastric antrum for histological examination and tissue Lf measurement. A *Helicobacter pylori* gastritis diagnosis was only made after histological examination of biopsy samples. Blood was taken from all cases for full blood count, serum iron, iron binding capacity (IBC), total iron binding capacity (TIBC), and ferritin level. Patients with a digestive system or systemic disorder other than Hp gastritis, cases previously treated for Hp gastritis or peptic ulcer disease, and patients taking proton pump inhibitors or antibiotics were not included in the study.

Tissue Lf level was evaluated by a blinded pathology specialist, who was not informed about the patients' clinical progress or the gastric antral immunohistochemical study results from primary antibody assays (DakoCytomation, LSAB2 System-HRP, Denmark).

Lactoferrin was quantitated immunohistochemically by two methods: in the first method, all samples were evaluated from 0% to 100% according to the staining percentage of glands in the antrum; in the second method, all samples were evaluated based on no staining (0), light (1), medium (2), or dense (3) according to stain-holding densities of glands in the antrum. Biopsy samples of cases were also grouped as no neutrophils (0), light (1), medium (2), or dense (3) according to the density of Lf-secreting neutrophils.

Data from this study were analyzed using the SPSS statistics software package. Nonparametric MannWhitney *U* and Chi-square tests were used to compare the data. In all tests, *P* < 0.05 was taken as significant.

## 3. Results

The study consisted of 64 patients with ages ranging between 4 and 17 years (average: 10.2 ± 0.4 years). 39 (60.9%) of the cases were male, while 25 (39.1%) were female. As a result of histological examination of the gastric antrum biopsy samples, 48 (75%) out of 64 cases were found to be Hp-positive, while 16 (25%) cases were Hp-negative. Endoscopic findings of Hp-positive and -negative cases are shown in Table 1. Antral nodularity in Hp-positive group was significantly higher compared to Hp-negative group (*P* = 0.008), (95% Confidence interval (CI) = 0.006–0.01).

 The average age of positive cases was 10.9 ± 0.5 years, and the average age of negative cases was 8.1 ± 0.9, which was statistically significant (*P* = 0.009). Positive Hp was seen in more male patients, but this difference was statistically insignificant (*P* = 0.1). (Table 2). Serum iron; IBC, TIBC, ferritin levels; full blood count and hemoglobin (Hb), hematocrit (Htc), leucocyte (WBC), thrombocyte (PLT), and erythrocyte count (RBC); average erythrocyte volume (MCV), average erythrocyte hemoglobin concentration (MCHC), erythrocyte distribution range (RDW), and average erythrocyte hemoglobin (MCH) values were also separately evaluated for Hp-positive and -negative cases, but no significant differences were observed. Results of power analyses for Mann-Whitney tests provided a wide range from very low to very high such as 0.051 for Hb, 0.386 for Htc, 0.421 for ferritin, and 0.948 for serum iron. These results can be interpreted as %39 power to detect a difference between Htc values of two groups with sample sizes of 16 and 48. (Table 3).

**Figure 1:**
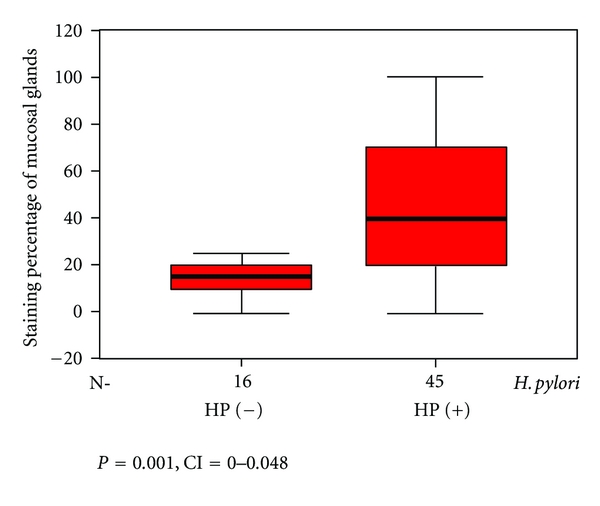
Staining percentage of antral mucosal glands by Hp-positive and -negative cases.

**Figure 2:**
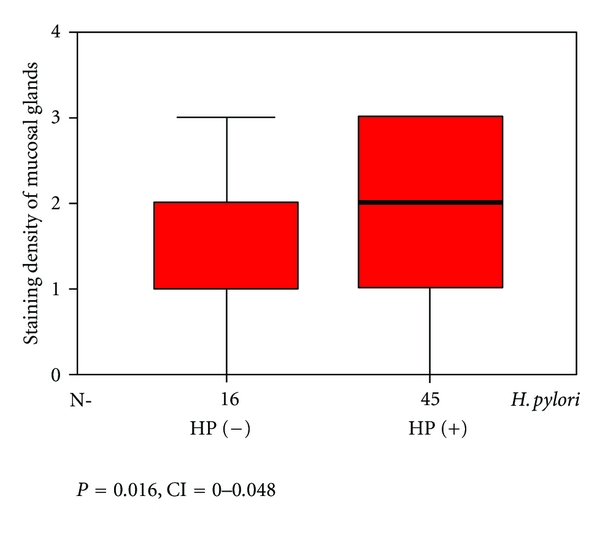
Staining density of antral mucosal glands by Hp-positive and -negative cases.

**Figure 3:**
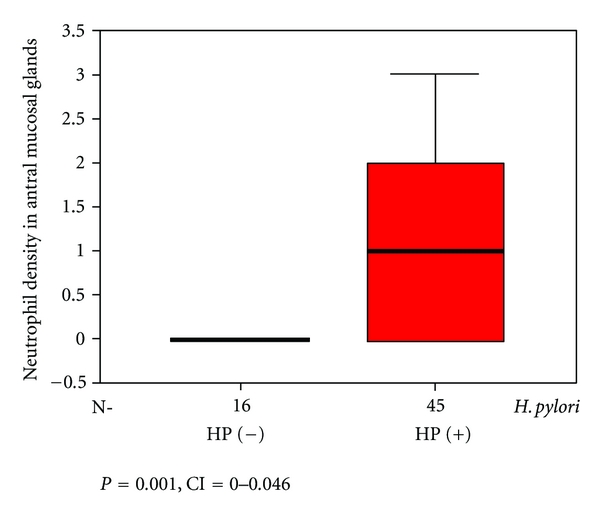
Neutrophil density in antral mucosal glands by Hp-positive and -negative cases.

As three cases had insufficient tissue for assay, tissue Lf was only measured in the remaining 61 cases. 45 of these cases were Hp-positive, while 16 were Hp-negative. In Hp positive cases, staining percentages and density of glands in the antral mucosa were 45.5 ± 4.7% and 1.9 ± 0.1, respectively, while these figures were 17.8 ± 4.5% and 1.3 ± 0.2 [(*P* = 0.001, CI = 0.0–0.048), (*P* = 0.016, CI = 0.0–0.048)] in Hp-negative cases. (Figures 1 and 2). The neutrophil density in the Hp-positive group was also significantly higher compared to the Hp-negative group. (*P* = 0.001, CI = 0.0–0.046) (Figure 3).

## 4. Discussion

One of the most important clinical findings caused by Hp infection other than gastrointestinal diseases is anemia [8]. There are quite a number of studies in the literature demonstrating the relationship between Hp and anemia in both adults and infants [9–12]. For example, in a study of young patients in South Korea, IDA in individuals infected with Hp was reported as 2.9 times higher compared to uninfected cases [13]. It has been reported that this anemia did not improve despite iron treatment, but the hemoglobin, serum iron, and ferritin levels improved following treatment of Hp infection [14]. In a random, placebo-controlled study in 43 infants and adults, hemoglobin levels were also reported to be significantly improved following Hp treatment [15].

In addition to studies suggesting a relationship between Hp and anemia, there are also studies suggesting the opposite. Collet et al. [16], in a study conducted on 1060 cases, reported that there was no significant difference between serum ferritin level and HP in both males and females. In a review of the current data, Bini [17] tried to analyze whether Hp was really to blame for anemia in these cases or whether it is an innocent bystander. He tried to find the answers as to why Hp causes anemia in only a small percentage of infected patients, how this organism causes anemia if at all, and whether or not there is improvement in anemia after treatment. As a result of this evaluation, it was shown that Hp positively correlated with low levels of ferritin, though this may not play a direct role. Our results were contrary to other studies that reported a relationship between Hp and IDA, as there was no significant difference between the average hemoglobin, hematocrit, serum iron, and ferritin levels of Hp-positive and Hp-negative groups (Table 3).

Despite all these results, it is still unclear with which mechanism Hp causes IDA in these cases. Mechanisms proposed include loss of blood from the digestive system, poor iron intake, iron absorption disorder, and use of iron in the reticuloendothelial system [11, 18, 19], and Hp is an etiological factor for gastric atrophy. Hypo- or achlorhydria may develop in individuals with atrophic gastritis, and iron absorption may decrease as a result [20]. Contrary to current theory, it has been proposed that Lf secreted from Hp-activated neutrophils in the stomach transfers iron to the outer cell wall receptor of Hp and that an IDA then results [6, 7]. Though not many studies support this theory, Nakao et al. [21] reported that there is a significant increase in Lf in the gastric fluids and mucosal tissue samples in Hp-infected patients.

Lonnerdal and Iyer [22] reported that the main cause of this Lf increase in gastric tissue was the increase in the inflammatory mediator interleukin-8 causing attraction of neutrophils and monocytes which then secrete Lf. A different study by Nakao et al. [23] also reported a marked relationship between interleukin levels in the gastric mucosa and Lf increase.

Choe et al. [24] reported a study on 101 patients with unexplained epigastric pain and/or iron deficiency, which found markedly high Lf levels in the Hp-positive and anemia group, and Lf levels in the Hp-positive group were found to be significantly raised relative to the control group and anemia-only group. In the same study, control endoscopy 8 weeks after Hp treatment of 12 of the Hp-positive cases with anemia measured Lf and hemoglobin levels in the gastric tissue biopsies. They reported a decrease in tissue Lf and a marked increase in hemoglobin compared to pretreatment.

In this study, staining percentage of antral gland cells of Hp-positive cases and staining density of cells were significantly higher compared to Hp-negative cases (*P* = 0.001, *P* = 0.016). Moreover, the percentage of stained neutrophils in Hp-positive cases was markedly higher compared to Hp-negative cases (*P* = 0.001).

The increase in Lf in Hp-positive cases agreed with previously conducted studies, but conversely, hemoglobin, hematocrit, and ferritin levels in these cases did not differ (Table 3). These results did not match the hypothesis that anemia is caused by loss of iron to Hp bacteria via Lf in the gastric tissue of Hp-positive cases.

We can conclude that the increase of Lf in the gastric tissue of Hp-positive cases is dependent upon inflammation. However, this iron does not seem to be transferred to Hp, as overall iron levels did not decrease in these patients and so we cannot say that the increase in Lf is connected with the subsequent development of anemia.

## Figures and Tables

**Table 1:** Endoscopic findings of Hp-positive and -negative cases.

	Hp-positive cases		Hp-negative cases		Total cases	
	*N*	%	*N*	%	*N*	%
Number of subjects	48	75	16	25	64	100
Endoscopic findings						
Normal	6	12.5	8	50	14	21.9
Antral hyperemia	15	31.2	6	37.5	21	32.8
Antral nodularity*	27	56.2	2	12.5	29	45.3

*P = 0.008 95%, confidence interval (CI): 0.006–0.01.

**Table 2:** Details of cases included in the study.

Characteristics	Hp-positive cases		Hp-negative cases		Total Cases		*P* value
	*N*	Mean ± SEM	*N*	Mean ± SEM	*N*	Mean ± SEM	
Mean (Age)	48	10.9 ± 0.5	16	8.1 ± 0.9	64	10.2 ± 0.4	0.009*
Male	32	10.5 ± 0.6	7	8.51.7	39	10.18 ± 0.6	0.1^†^
Female	16	11.8 ± 0.8	9	7.8 ± 0.9	25	10.43 ± 0.7	
Weight (kg)	48	35.63 ± 1.85	16	27.46 ± 2.77	64	33.58 ± 1.6	0.03*
Height (cm)	48	139.99 ± 2.78	16	128.56 ± 4.84	64	137.13 ± 2.4	0.056^†^

*P < 0.05.

^†^ *P* > 0.05.

SEM: Standard. error of mean.

**Table 3:** Full blood count, serum iron, TIBC, and ferritin values of cases.

	Hp-positive	Hp-negative	*P* value
Hb (gr/dL)	12.6 ± 0.1	12.6 ± 0.2	0.7
Htc (%)	37.3 ± 0.4	37.0 ± 0.8	0.7
WBC (mm^3^)	7203 ± 258	7836 ± 690	0.2
PLT (mm^3^)	303520 ± 12048	282187 ± 17604	0.3
RBC (mm^3^)	4526479 ± 47995	4565625 ± 92812	0.6
MCV	82.0 ± 0.7	80.6 ± 1.4	0.3
MCHC	34.0 ± 0.2	33.8 ± 0.2	0.6
RDW	12.6 ± 0.1	12.9 ± 0.2	0.1
MCH	28.5 ± 0.2	27.6 ± 0.5	0.1
Serum Iron	72.9 ± 5.3	57.5 ± 5.7	0.1
IBC	274.1 ± 8.8	274 ± 7.5	0.9
TIBC	347 ± 7.2	331.5 ± 7.3	0.2
Ferritin	32.3 ± 2.4	30.7 ± 4.4	0.7
